# Application of MR-ProADM to predict prevention of hospitalisation, derived from a multi-centre study

**DOI:** 10.1186/s13054-019-2402-0

**Published:** 2019-04-17

**Authors:** Milo Cullinan, Sara Graziadio, David Ashley Price

**Affiliations:** 1 Laboratory Medicine, Newcastle-upon-Tyne Hospitals Foundation Trust, Queen Victoria Road, Newcastle-upon-Tyne, NE1 4LP UK; 2 NIHR Newcastle In Vitro Diagnostics Co-operative, Newcastle-upon-Tyne Hospitals Foundation Trust, Queen Victoria Road, Newcastle-upon-Tyne, NE1 4LP UK

Dear editor,

We read with interest the paper presented by Saeed et al [1], ‘The early identification of disease progression in patients with suspected infection presenting to the emergency department: a multi-centre derivation and validation study’. We retrospectively applied the cut-offs for mid-regional pro-adrenomedullin (MR-proADM) and C-reactive protein (CRP) derived in this study to the cohort used in our paper, ‘Can mid-regional pro-adrenomedullin (MR-proADM) increase the prognostic accuracy of NEWS in predicting deterioration in patients admitted to hospital with mild to moderately severe illness? A prospective single-centre observational study’ [2] to see if we could replicate the sensitivity and specificity obtained for predicting avoidance of hospitalisation, defined as a discharge to community care within 24 h of presentation.

Our original study was a prospective observational cohort study [2]. Between September and December 2015, 300 patients who attended the Medical Assessment Unit at the Royal Victoria Infirmary, Newcastle, were enrolled. Patients were included whose National Early Warning Score (NEWS) was greater than or equal to 2 and less than or equal to 5. Those admitted for purely social reasons, were to receive only palliative care or had been admitted due to self-harm or overdose were excluded.

One hundred twenty-three patients had, at discharge, a diagnosis of an infection. Twenty (16.3%) of these were discharged within 24 h. An MR-proADM of less than 0.87 nmol L^−1^ gave a sensitivity of 60% (44–71% 95%CI) and a specificity of 60% (53–66% CI) for discharge within 24 h. See Table 1 for comparison to Saeed et al. [1].

**Table 1:** Test accuracy for prediction of hospitalisation avoidance by Saeed et al. and from our data

		Graziadio et al [2]	Saeed et al [1]
MR-proADM	Sensitivity	0.60 [0.44–0.71]	0.75 [0.72–0.78]
	Specificity	0.60 [0.53–0.66]	0.80 [0.74–0.84]
	LR+	1.24 [0.82–1.86]	3.69 [2.90–4.71]
	LR−	0.78 [0.44–1.37]	0.31 [0.27–0.35]
	PPV	0.19 [0.14–0.87]	0.93 [0.91–0.95]
	NPV	0.87 [0.79–0.92]	0.48 [0.43–0.52]
CRP	Sensitivity	0.85 [0.62–0.97]	0.42 [0.39–0.45]
	Specificity	0.21 [0.13–0.30]	0.77 [0.72–0.82]
	LR+	1.07 [0.87–1.32]	1.84 [1.45–2.33]
	LR−	0.72 [0.24–2.19]	0.75 [0.69–0.82]
	PPV	0.18 [0.15–0.21]	0.87 [0.83–0.89]
	NPV	0.88 [0.70–0.96]	0.27 [0.24–0.31]

*MR-proADM* mid-regional pro-adrenomedullin, *CRP* C-reactive protein, *PPV* positive predictive value, *NPV* negative predictive value, *LR+* positive likelihood ratio, *LR***−** negative likelihood ratio

We have failed to replicate the utility suggested in the paper by Saeed et al. for MR-proADM predicting hospitalisation avoidance. However, the cohort here differs from that of Saeed et al. in many respects. Patients who attended the Medical Assessment Unit had already been assessed by either an emergency department physician or a general practitioner and have been considered to be likely to require hospitalisation. Furthermore, our study population was limited to those with NEWS score between 2 and 5 compared to the unselected cohort of Saeed et al. However, with a median NEWS of 4, their cohort’s NEWS may not differ greatly from ours.

This demonstrates that other patient characteristics and the stage in the patient pathway are likely to be important when choosing how to implement this biomarker in aiding decisions about avoiding hospitalisation. Earlier use of the MR-proADM in unselected patients in an ED setting may help select patients for early discharge.

## Abbreviations

CRP: C-reactive protein
LR−: Negative likelihood ratio
LR+: Positive likelihood ratio
MR-ProADM: Mid-regional proadrenomedullin
NEWS: National Early Warning Score
NPV: Negative predictive value
PPV: Positive predictive value
